# Improved biomass and protein production in solid-state cultures of an *Aspergillus sojae* strain harboring the *Vitreoscilla* hemoglobin

**DOI:** 10.1007/s00253-015-6851-3

**Published:** 2015-07-30

**Authors:** Rodrigo Mora-Lugo, Marvin Madrigal, Vikas Yelemane, Marcelo Fernandez-Lahore

**Affiliations:** 1 Downstream Bioprocessing Lab, Jacobs University Bremen gGmbH, Bremen, Germany; 2 Instituto Clodomiro Picado and Departamento de Bioquímica, Universidad de Costa Rica, San José, Costa Rica

**Keywords:** *Agrobacterium tumefaciens-*mediated transformation, *Aspergillus sojae*, Biomass production, Pectinases and protease, Solid-state fermentation, *Vitreoscilla* hemoglobin

## Abstract

The biotechnological value of *Aspergillus sojae* ATCC 20235 (*A. sojae*) for production of pectinases in solid-state fermentation (SSF) has been demonstrated recently. However, a common drawback of fungal solid-state cultures is the poor diffusion of oxygen into the fungi that limits its growth and biological productivity. The bacterial *Vitreoscilla* hemoglobin (VHb) has favored the metabolism and productivities of various bacterial and yeast strains besides alleviating hypoxic conditions of its native host, but the use of VHb in filamentous fungi still remains poor explored. Based on the known effects of VHb, this study assessed its applicability to improve *A. sojae* performance in SSF. The VHb gene (*vgb*) under control of the constitutive *Aspergillus nidulants gpdA* promoter was introduced into the genome of *A. sojae* by *Agrobacterium*-mediated transformation. Successful fungal transformants were identified by fluorescence microscopy and polymerase chain reaction (PCR) analyses. In solid-state cultures, the content of protease, exo-polygalacturonase (exo-PG), and exo-polymethylgalacturonase (exo-PMG) of the transformed fungus (*A. sojae* vgb+) improved were 26, 60, and 44 % higher, respectively, in comparison to its parental strain (*A. sojae* wt). Similarly, biomass content was also 1.3 times higher in the transformant strain. No significant difference was observed in endo-polygalacturonase (endo-PG) content between both fungal strains, suggesting dissimilar effects of VHb towards different enzymatic productions. Overall, our results show that biomass, protease, and exo-pectinase content of *A. sojae* in SSF can be improved by transformation with VHb.

## Introduction

Protein production by means of solid-state fermentation (SSF) has gained a lot of interest in the last years because it has many advantages over the most widely used submerged fermentation (SmF). For instance, the possibility to use low-cost substrates is a common benefit in solid-state cultures. Also, higher productivities, simpler equipment, and less space requirements are advantages associated to SSF (Aguilar et al. 2008; De la Cruz et al. 2014; Viniegra-Gonzalez et al. 2003). However, a major drawback in solid-state cultures with aerobic microorganisms is the lower diffusion of oxygen in the biomass which, in turn, limits the production of proteins (Stark et al. 2011; Wei and Chen 2008). To overcome oxygen limitations in fermentation processes, the co-expression of globins as a strain improvement strategy has proven to be useful in several aerobic hosts, including the filamentous fungi *Aspergillus oryzae* (Stark et al. 2011; te Biesebeke et al. 2006).

In various examples with prokaryotic and eukaryotic microorganisms, heterologous expression of the bacterial *Vitreoscilla* hemoglobin (VHb) has improved cell growth and protein synthesis under oxygen-limiting conditions most likely by enhancing respiratory metabolism (Stark et al. 2011, 2015; Wei and Chen 2008). VHb is the best-characterized member of the bacterial hemoglobin proteins, and since the identification of its amino acid sequence (Wakabayashi et al. 1986), its application for strain improvement in various organisms has been widely explored. Mostly in bacterial and yeast bioprocesses, cell growth, bioremediation, and enhanced protein production have been improved within this so called “*vgb/*VHb technology” (Hofmann et al. 2009; Kahraman et al. 2011; Kahraman and Erenler 2012; Wu and Fu 2012; Zhu et al. 2011). However, the potential of VHb has not been investigated in detail​ in filamentous fungi, mainly due to the availability of fewer genetic molecular tools to engineer them in comparison to bacterial and yeast hosts (Fleissner and Dersch 2010; Mora-Lugo et al. 2014; Ward 2012).

The potential of the filamentous fungus *Aspergillus sojae* ATCC 20235 (*A. sojae*) for production of pectinases by fermentative processes has been demonstrated in the last years (Ustok et al. 2007; Tari et al. 2008; Demir et al. 2012; Heerd et al. 2014a). Pectinases or pectinolytic enzymes are a heterogeneous group of related enzymes that hydrolyze pectic substances or pectins and are valuable biocatalysts for food and industrial applications (Adapa et al. 2014). *A. sojae* has yielded higher amounts of pectinases in comparison with the well-known pectinase producer *Aspergillus niger* and other *A. sojae* strains (CBS 100928 and IMI 191303) in SSF (Heerd et al. 2012). Moreover, through classic mutagenesis screening strategies based on physical (ultraviolet irradiation) and chemical mutagens, pectinase titers of descending mutants of *A. sojae* have been improved in SmF and SSF (Heerd et al. 2014b). The potential of some of these high-yield pectinase mutants has been further explored by measuring various pectinase activities including exo-/endo-polygalacturonase (exo-PG/endo-PG), exo-polymethylgalacturonase (exo-PMG), and pectin lyase using two different carbon sources in SSF (Mata-Gomez et al. 2014). Recently, an *Agrobacterium tumefaciens*-mediated transformation (ATMT) method was described for *A. sojae*, and heterologous expression of the enhanced green fluorescent protein (EGFP) was demonstrated successfully (Mora-Lugo et al. 2014). This study opens up new possibilities of protein expression studies in *A. sojae* and explore systematically strain improvement strategies for this fungus based on genetic molecular tools.

Based on the positive effects of VHb in previous microbial hosts, this study describes a genetic engineering approach to improve *A. sojae* for SSF. This fungus was genetically engineered with VHb through an adapted transformation method mediated by *A. tumefaciens*. Subsequently, different pectinases, protease, and biomass content were measured and compared between the transformed fungus (*A. sojae*vgb+) and its parental strain (*A. sojae* wt) in solid-state cultures. The present study provides a new strain improvement strategy for *A. sojae* to further explore its potential as pectinase biofactory.

## Material and methods

### Materials

All chemicals were purchased from AppliChem GmbH (Darmstadt, Germany), except citrus pectin, galacturonic acid, polygalacturonic acid sodium salt, and D-(+)-glucosamine hydrochloride were obtained from Sigma-Aldrich Chemie GmbH (Steinheim, Germany). Microbial substrates like wheat bran, sugar beet pulp pellets, and molasses were obtained from local suppliers (Bremer Rolandmühle Erling GmbH & Co. KG, Bremen, Germany; Nordzucker AG, Uelzen, Germany; Golden Sweet, Meckenheim, Germany). Restriction enzymes, T4 DNA polymerase, and T4 DNA ligase were purchased from New England Biolabs (Frankfurt am Main, Germany). Oligoprimers and DNA sequencing services were ordered from Eurofins (Ebersberg, Germany).

### Microorganisms and media

*A. sojae* ATCC 20235 was grown at 28 °C until conidiation (3–6 days) on molasses agar plates (45 g/l molasses, 45 g/l glycerol, 18 g/l peptone, 5 g/l NaCl, 0.5 g/l KCl, 15 mg/l FeSO_4_ · 7H_2_O, 60 mg/l KH_2_PO_4_, 50 mg/l MgSO_4_, 12 mg/l CuSO_4_ · 5H_2_O, 15 mg/l MnSO_4_ · H_2_O, and 20 g/l agar). Spores were harvested using 0.02 % (*w*/*v*) Tween 80 and filtered through cotton to remove hyphae. The spore concentration was determined using a Neubauer chamber (Celeromics, Grenoble, France). Top 10 *Escherichia coli* cells (Invitrogen, CA, USA) were used as a host for all DNA manipulations. DNA plasmids were isolated from Luria-Bertani overnight cultures supplemented with 100 μg/ml streptomycin and 50 μg/ml kanamycin, using the “NucleoSpin Plasmid” commercial kit (Macherey-Nagel, Düren, Germany). *Agrobacterium tumefaciens*, strain LBA4404 (ElectroMAX^™^, Invitrogen, CA, USA), was used as donor of the transfer DNA (T-DNA) for fungal transformation of *A. sojae*.

### Construction of *vgb*-gene donor vector and fungal transformation

**Fig. 1:**
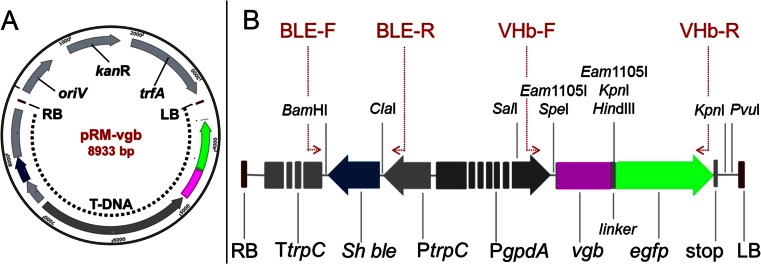
**a** The vector pRM-vgb was used for ATMT of *A. sojae* and contains the kanamycin resistance gene (*kan*R), trans-acting factor A gene (*trfA*), replication origin (*oriV*), and transfer DNA region (T-DNA). **b** Enlargement of the T-DNA region that is delimited by the right and left border (*RB* and *LB*, respectively) and contains the *Vitreoscilla* hemoglobin gene (*vgb*) and the reporter enhanced green fluorescent protein gene (*egfp*) under control of the constitutive *Aspergillus nidulants gpdA* promoter (P*gpdA*), the phleomycin resistance gene from *Stretoalloteichus hindustanus* (*Sh ble*) under control of the *A. nidulants* trpC promoter (P*trpC*) and trpC terminator (T*trpC*). The *red arrows* indicate the target sites for the oligonucleotide primers BLE-F/R, VHb-F/R. Target sites for restriction enzymes are also shown

The ATMT donor vector named pRM-vgb (GenBank Accession No KT225581) was designed for cloning and expression of the *vgb* gene under control of the constitutive *Aspergillus nidulants gpdA* promoter (P*gpdA*) in *A. sojae* (Fig. 1). This vector was derived from the pRM-eGFP vector (Mora-Lugo et al. 2014) by subcloning a 1.7-kb synthetic cassette (ordered from Eurofins, Ebersberg, Germany) between *Sal*I and *Pvu*I restriction sites. This cassette contains *vgb*, which was codon usage optimized for *Aspergillus* species according to the CUTG database (GenBank) and fused to the 5′ end of the reporter *egfp* gene. Moreover, other additional restriction sites for forthcoming cloning strategies were added in this operon including two *Kpn*I to enable deletion of *egfp*, if required, and two *Eam*1105I to allow TA cloning (by 3′T overhanging ends) for gene expression under control of the P*gpdA* (Alibu et al. 2005). The constructed pRM-vgb vector was electro-transformed into *A. tumefaciens* LBA4404, and recombinant bacteria were selected on LB agar plates containing 100 μg/ml streptomycin and 50 μg/ml kanamycin. Accuracy of the plasmid sequence was examined by restriction enzyme digestion and sequencing analysis. The recombinant *A. tumefaciens* strain containing the pRM-vgb vector was used to transform *A. sojae* by the ATMT procedure as described by Mora-Lugo et al. (2014).

### Analysis of the fungal transformants

Putative *A. sojae* transformants were assessed by PCR and fluorescence microscopy analysis to verify chromosomal integration and gene expression of the transformed or transferred DNA (T-DNA), respectively, as described by Mora-Lugo et al. (2014). For the PCR analysis, the genomic DNA from putative fungal transformants was isolated and used as DNA template. The specific primers sets BLE-F/BLE-R and VHb-F/VHb-R (Table 1) were used to target the *ble* and *vgb* genes located in the T-DNA region, respectively. Amplification included an initial denaturation at 94 °C for 5 min, followed by 30 cycles of denaturation at 94 °C for 15 s, annealing for 40 s (at 55 °C for *ble* and at 59 °C for *vgb*), and elongation at 68 °C for 60 s, and a final elongation step at 68 °C for 5 min. Genomic DNA from the parental *A. sojae* wt strain and purified pRM-vgb plasmid were used as a negative and positive control, respectively. For the fluorescence microscopy analysis, the expression of EGFP reporter gene in *A. sojae* transformants was visualized using an Axioplan 2 imaging (Zeiss) microscope equipped with a filter set matching the excitation and emission spectra for the EGFP (Ex/Em = 488/509). Images were acquired with the AxioVision (*V. 4.8*) software, setting an exposure time of 900 ms to subtract the background autofluorescence signal of the *A. sojae* wt.

**Table 1:** Sequences of the primers used for PCR amplification

Primer name	Primer sequence	Reference
BLE-F	5′-CGTTTTATTCTTGTTGACATGGAGC-3′	Mora-Lugo et al. (2014)
BLE-R	5′-TTGGGCTTGGCTGGAGCTAGTGGAG-3′	
VHb-F	5′-CAGTTCGAGCTTTCCCACTTCATCG-3′	This study
VHb-R	5′-TGTACAGCTCGTCCATGCCGAGAGT-3′	

### Solid-state fermentation at flask scale

SSF experiments were carried out independently, in triplicates, and for 10 days with an untransformed (*A. sojae* wt) and a selected transformed fungal strain (*A. sojae* vgb+) according to the method provided by Heerd et al. (2014b) with slight modifications. Erlenmeyer flasks (300 ml) containing 10 g of wheat bran and ground sugar beet pulp in the ratio 70:30 and wetted at 160 % with 16 ml of 0.2 M HCl solution were sterilized at 121 °C for 20 min. Each flask was inoculated with a total number of 2 × 10^7^ fungal spores and incubated at 30 °C. A non-inoculated flask was used as a control sample blank. A replicate for each fungal strain consisted of ten individual flasks, and one of each was used once every day for sampling. Fermentation samples were collected by adding 80 ml distilled water per flask, homogenized partially with a spatula, and mixed in an incubator shaker (Innova 4230, New Brunswick Scientific) at 250 rpm and 25 °C for 1 h. The supernatants were clarified by centrifugation at 3200 × *g* and 4 °C for 20 min and filtration through Whatman #1 (11 μm pore size) and set aside for protein content analyses. The precipitated pellets (wet fermented substrate) were lyophilized for 2 days with freeze dryer (Alpha 1-2/LD plus, Christ, Osterode am Harz, Germany), ground to a fine powder with mortar and pestle, and set aside for glucosamine content (fungal biomass analyses).

### Protein production

Different protein contents (exo-PG, exo PMG, endo-PG, and protease) were measured from the clarified extracts and expressed as enzymatic activity unit per gram of substrate (U/g). The data obtained were represented as mean ± SD.

### Exo-polygalacturonase and exo-polymethylgalacturonase

Exo-PG and exo-PMG activities were measured according to previously described protocols (Blandino et al. 2002; Heerd et al. 2012; Silva et al. 2005) with slight modifications. The assays were performed in a microplate by mixing 10 μl of enzymatic sample and 90 μl of 5 μg/μl of substrate (polygalacturonic acid (PGA) for exo-PG activity and citrus pectin for exo-PMG activity) in a 100 mM citrate-Na biphosphate buffer pH 5.0. The reduced galacturonic acid (GA) released by the reaction after 30 min of incubation at 30 °C for exo-PG activity and 10 min of incubation at 45 °C for exo-PMG activity was quantified by the DNS method at 575 nm (Miller 1959) and compared to a GA standard curve. One unit of exo-enzyme activity was defined as the amount of enzyme that catalyzes the release of 1 μmol of GA per minute at the standard assay conditions mentioned above.

### Endo-polygalacturonase

Endo-PG activity was measured according to an adapted method from (Ortiz et al. 2014). The assay was performed in a microplate by mixing 8 μl of enzymatic sample and 8 μl of 5 μg/μl PGA substrate in a 100 mM citrate-Na biphosphate buffer pH 5.0. The hydrolyzed PGA unable to precipitate with ruthenium red dye (RR) after 20 min of reaction at 40 °C was measured at 535 nm and compared to a PGA standard curve (from 0 to 36 μg). One unit of enzyme activity was defined as the amount of enzyme required to hydrolyze 1 μg of PGA in smaller fragments unable to precipitate with RR per minute at the standard assay conditions mentioned above.

### Protease

Protease activity was measured according to the commercial Pierce protease assay kit (Thermo Scientific, Illinois, USA). The assay was performed in a microplate by mixing 50 μl of enzymatic sample and 100 μl of 2 μg/μl succinylated casein (substrate) in a 50 mM borate buffer pH 8.5. The released tyrosine (product) by the reaction after 20 min of incubation at 37 °C was measured at 450 nm and compared to a TPCK-trypsin standard curve. One unit of protease activity was defined as the amount of enzyme that converts as much substrate as 1 μg of TPCK-trypsin (standard protease) per minute at the standard assay conditions mentioned above.

### Total soluble protein

Total soluble protein was measured in SSF-supernatant samples according to the modified Bradford method (Bradford 1976) from the commercial Coomassie Plus™ Protein Assay Kit (Thermo Scientific, Illinois, USA). The assay was performed using the standard microplate protocol and bovine serum albumin (included in the kit) as a standard. Absorbance values were measured at 595 nm.

### Biomass production

The glucosamine (GlcN) released by acid hydrolysis of chitin present in the cell wall of the filamentous fungi was measured as an indirect method to estimate biomass content in the fermented samples. GlcN content in samples of 0.1 g of dried fermented substrate (dfs) was assessed according to Zamani et al. (2008) with slight modifications and compared to a standard curve of D-(+)-glucosamine hydrochloride. Fungal biomass was expressed in terms of milligram of GlcN (mg_GlcN_) per gram of dfs (from here on referred just as mg/g). The data obtained were represented as mean ± SD.

## Results

### Cloning of *vgb* gene in *A. sojae*

The VHb gene (*vgb*) was transformed into *A. sojae* ATCC 20235 following the protocol described by Mora-Lugo et al. (2014). *A. tumefaciens* LBA4404 containing the vector pRM-vgb (Fig. 1a) was utilized for chromosomal integration of the T-DNA element containing *vgb* as well as the *ble* gene for phleomycin resistance (Fig. 1b). *A. sojae* transformants were selected from minimal medium agar plates containing 50 μg/ml phleomycin. After 4 days of incubation, eight fungal colonies per 10^5^ conidia were obtained from the selection plates. These putative transformants were subcultured individually on PDA plates supplemented with 100 μg/ml phleomycin for four generations to evaluate the stability of the T-DNA that confers antibiotic resistance. Three out of eight transformants survived after the antibiotic treatment and were used for further analysis.

### Verification of transformation using PCR

**Fig. 2:**
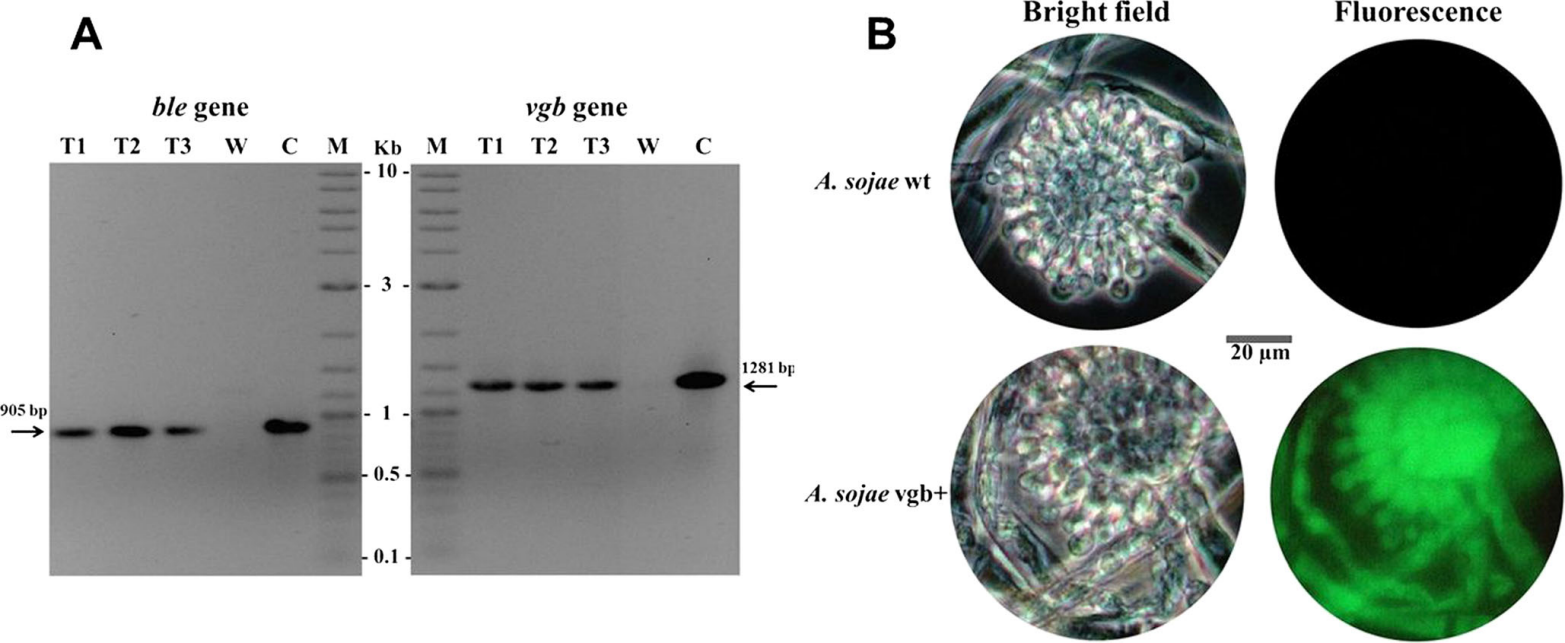
**a** Verification of putative *A. sojae* transformants by PCR analysis. The amplified PCR products at the expected size (highlighted with *arrows*) confirm the presence of the *ble* gene and the *vgb* gene in the genomic DNA samples of the fungal transformants. Genomic DNA of *A. sojae* wt was used as negative (*W)* and purified pRM-vgb vector as positive control (*C*); molecular size marker (*M*). **b** Verification of *egfp* expression in the selected *A. sojae* vgb + transformant by fluorescence microscopy analysis. *A. sojae* wt was used as negative control

The three obtained phleomycin-resistant transformants were examined by PCR analysis to confirm successful chromosomal integration of the T-DNA cassette. Genomic DNA from these strains and their parental strain (*A. sojae* wt) as negative control were used to target the *ble* and *vgb* genes by PCR with the specific primers sets BLE-F/R and VHb-F/R (Table 1), respectively. PCR products of expected sizes of 905 bp for *ble* and 1281 bp for *vgb* were obtained from the transformants while in the wild-type sample, these DNA regions were absent (Fig. 2). Bidirectional sequencing of the PCR products with the primers used for PCR confirmed the identity of *ble* and *vgb* and thus successful integration of these genes into the genome of the *A. sojae* transformants.

### Verification of *egfp* expression using fluorescence microscopy

Additionally to the presence of the *egfp* reporter gene by PCR analysis, expression of the gene in the fungal transformants was verified by fluorescence microscopy analysis. Fluorescence signals were observed in the fungal transformants containing *vgb* and *egfp* in the same operon. In contrast, we observed no fluorescence signal in the parental *A. sojae* wt (Fig. 2). Fluorescence microscopy analysis facilitated further control and discrimination between transformed and untransformed fungus as there was no noticeable morphological difference between these fungal colonies.

After PCR and fluorescence microscopy analysis, the three positive fungal transformants were screened on agar plates supplemented with PGA to determine variation of pectinolytic activity between themas described by Martos et al. (2013). As there was neither difference in the zones of pectin hydrolysis on the agar plates nor phenotypical changes observed between the fungi, one of the positive transformants was randomly selected (*A. sojae* vgb+) to carry out all the subsequent SSF experiments.

### SSF with the transformed and untransformed fungus

**Fig. 3:**
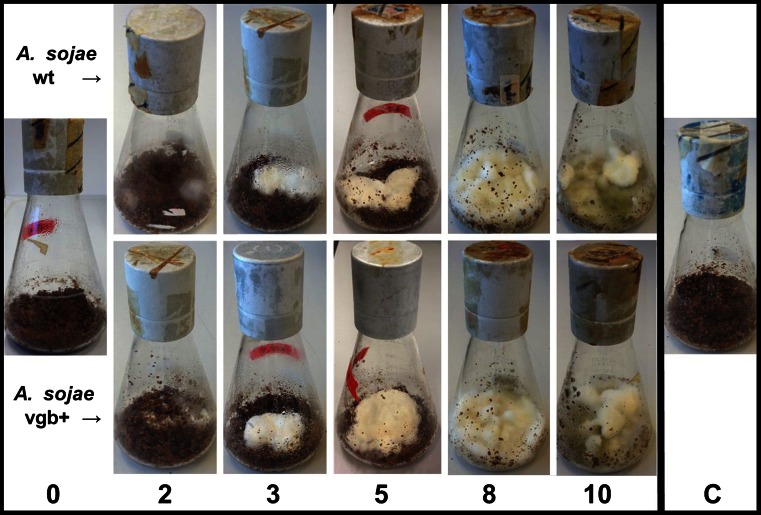
Typical growth of *A. sojae* wt and *A. sojae* vgb + on solid substrate during the 10-day fermentation period (only selected days are shown). The *column on the left* shows a representative flask of the inoculation day (0), and the *column on the right* shows the non-inoculated control media after 10 days of incubation under the same conditions (*C*)

The growth of the transformant *A. sojae* vgb + and parental *A. sojae* wt strain in the solid-state cultures was visually examined. Both fungal strains showed a similar grow pattern during each of the entire fermentation period of 10 days (Fig. 3). The first mold growth was observable 2 days after inoculation, and mycelia were patchily distributed on the substrate. Abundant mycelial growth was observed after 4 days of fermentation with total colonization of the solid substrate by day 6. The first conidia production was observed after 6 to 7 days of fermentation reaching its maximum content by the end of the fermentation at day 10, where conidia are indicated by the green color (Fig. 3).

### Production of extracellular proteins

**Fig. 4:**
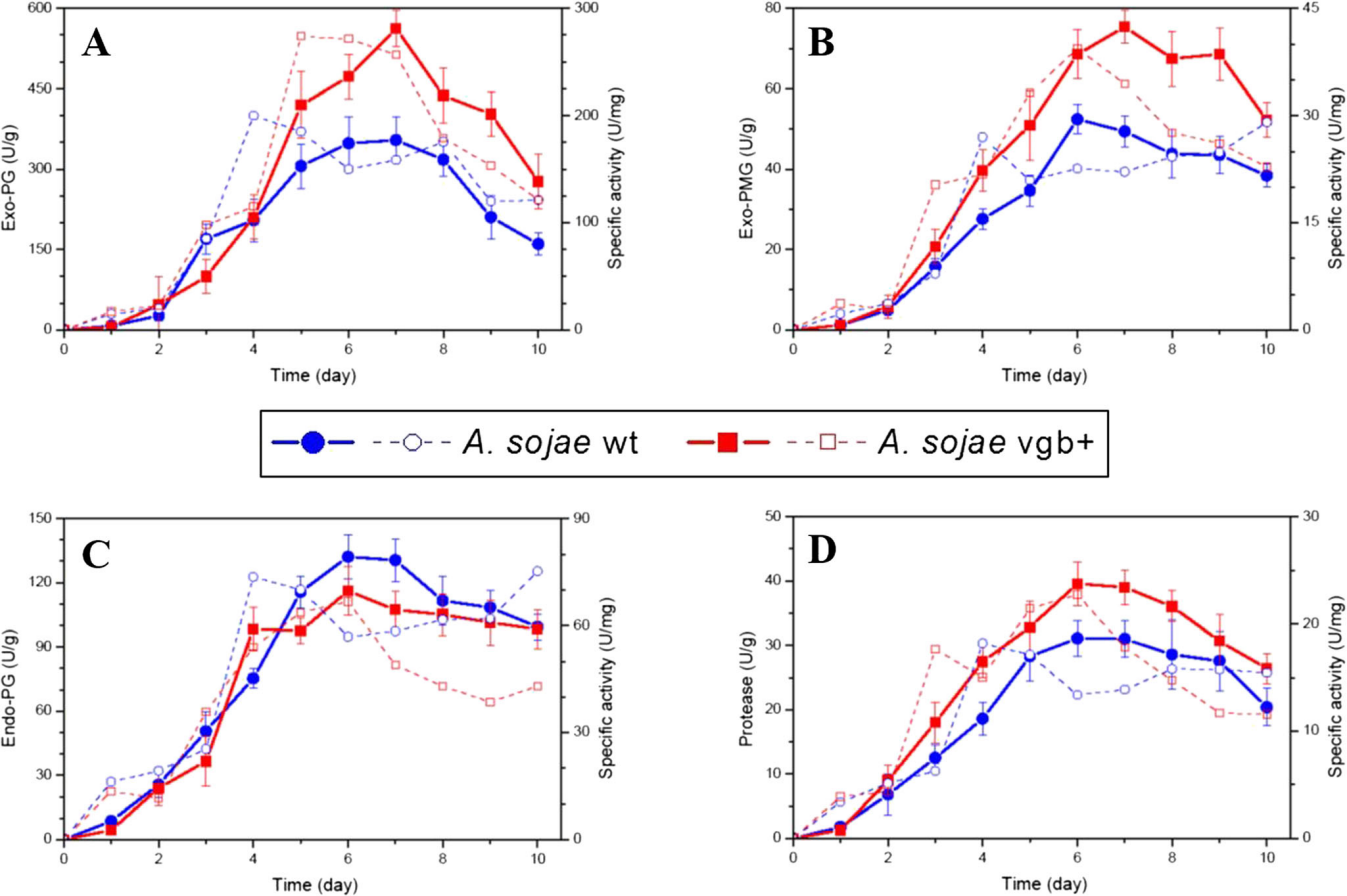
Protein production of *A. sojae* wt and *A. sojae* vgb + in SSF. Exo-PG (**a**), exo-PMG (**b**), endo-PG (**c**), and protease (**d**) content were determined from enzymatic extracts collected every 24 h during the 10-day incubation period. *Solid lines* indicate the enzymatic yield, and *dashed lines* indicate specific activity. Each data point represents the average ± SD from fermentations carried out in triplicates

To evaluate whether VHb had a positive effect on the production of extracellular proteins of *A. sojae* in SSF, fermented samples of the transformant *A. sojae* vgb + and parental *A. sojae* wt strain were harvested daily and assessed for various enzymatic activities. A clear difference in protein production was observed between both fungal strains (Fig. 4). Regarding to pectinase production, the maximum exo-PG and exo-PMG titers of 562.1 and 75.4 U/g after 7 days of fermentation with *A. sojae* vgb + were nearly 1.60-fold and 1.45-fold higher, respectively, in comparison to the wild-type strain (Fig. 4a–b). However, no relative increase of endo-PG content was measured in the recombinant fungus, compared to the wild-type strain. In this case, the maximum endo-PG titer of 132.1 U/g was observed for the wild-type strain after 6 days of fermentation (Fig. 4c). Regarding protease production, the maximum quantity of 39.2 U/g was nearly 1.25-fold higher in the transformed fungus compared to the parental strain after 6 days of fermentation (Fig. 4d). Overall, the maximum enzymatic titers for both fungal strains were between the sixth and seventh day of fermentation. The pH of all enzymatic extracts increased overtime ranging from pH 3.9 ± 0.3 at the start to pH 5.8 ± 0.5 at the end of the fermentation, in both the transformant and wild-type strains.

### Biomass production

**Fig. 5:**
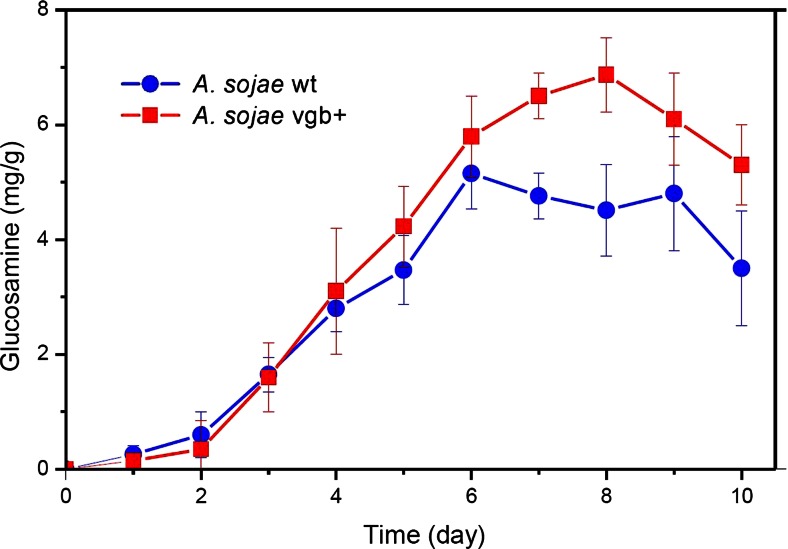
Biomass content of *A. sojae* wt and *A. sojae* vgb + in SSF. The values plotted are expressed as milligram of glucosamine (mg_GlcN_) per gram of dried fermented substrate (dfs), which were collected every 24 h during the 10-day incubation time. Each data point represents the average ± SD from fermentations carried out in triplicates

The production of biomass of *A. sojae* wt and *A. sojae* vgb + on the solid-state cultures was indirectly evaluated by measuring the glucosamine content of the cell wall of the fungi. In general, biomass titers were higher in the transformed fungus in comparison to the wild-type cultures, with significantly improved levels between the seventh and tenth day of fermentation (Fig. 5). The maximum glucosamine content of 6.9 mg/g measured in the recombinant fungus after 8 days of fermentation was 1.33-fold higher compared to the maximum content of the parental strain after 6 days of fermentation. Such improvement of biomass content in *A. sojae* vgb + was similar in order of magnitude to its improved levels of extracellular protein such as protease and exo-pectinases.

**Fig. 6:**
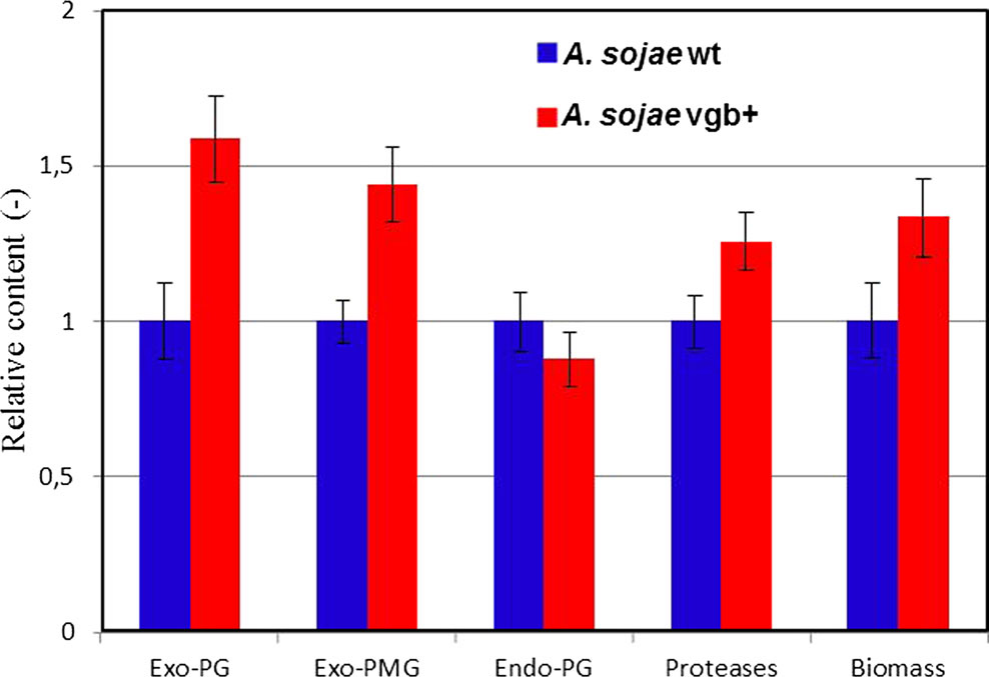
Relative yields of *A. sojae* wt and *A. sojae* vgb + in SSF. The maximum yields of the parental *A. sojae* wt strain were adjusted to 1.0 and compared with the normalized maximum titers of the transformed *A. sojae* vgb + strain

Figure 6 summarizes the various enzymatic and biomass maximum yields for both fungal strains, where standard errors never exceeded 10 %.

## Discussion

*A. sojae* ATCC 20235 has demonstrated recently its potential for pectinase production in fermentation systems and particularly in SSF (Demir et al. 2012; Gogus et al. 2006; Heerd et al. 2014a, 2014b, 2012; Mata-Gomez et al. 2014; Tari et al. 2008, 2007; Ustok et al. 2007). However, it is generally assumed that there is a limitation in the oxygen supply to the cells that are in close contact with the substrate in solid-state cultures with filamentous fungi (Oostra et al. 2001; Rahardjo et al. 2005). Previous studies have demonstrated beneficial effects of VHb to alleviate hypoxic conditions in several bacteria and yeast hosts (various summarized in Wei and Chen 2008; Stark et al. 2011, and Stark et al. 2015). Thus, this study was aimed to alleviate hypoxic conditions for *A. sojae* in SSF by genetically engineering this fungus with the VHb in order to improve its cell growth and protein production levels. The effect of this hemoglobin in *A. sojae* was not explored until now, and this so called *vgb/*VHb technology has rarely been used for SSF applications.

The VHb gene under control of the constitutive *A. nidulants gpdA* promoter (P*gpdA*) was integrated into the genome of *A. sojae* by an adapted method mediated by *A. tumefaciens* (ATMT). This transformation method was selected as its applicability for this fungus was previously demonstrated (Mora-Lugo et al. 2014). Likewise, chromosomal integration of the transformed T-DNA cassette and expression of the reporter EGFP was successfully confirmed, indicating the presence of the protein product of *vgb* in the fungal transformants (Fig. 2). However, about ten times fewer putative transformants (eight fungal colonies per 10^5^ conidia) were obtained in comparison to the previous study. A 482-bp larger T-DNA fragment was cloned in this study, which may have resulted in a lower number of transformants. Similarly, it is known from other studies on fungi, bacteria, and plant cells that increasing DNA fragment size can result in lower transformation efficiencies (Fleming et al. 1995; Gouka et al. 1999; Kung et al. 2013). Nevertheless, the genetic stability of the T-DNA cassette in the transformants was comparable to the mitotic stability rate of 40 % previously reported (Mora-Lugo et al. 2014). Our results demonstrate thus that even though transformation efficiency was relatively low, mitotically stable transformants can be obtained by the adapted ATMT method and indicate the applicability of this approach to explore the *vgb*/VHb technology on *A. sojae* for fermentation experiments.

Our results also show that *A. sojae* harboring the *vgb* gene (*A. sojae* vgb+) yields higher amounts of biomass, protease, and exo-pectinases in comparison to its parental strain *A. sojae* wt in SSF (Figs. 4–6). The results are in good agreement with recent reports of fungi, where increased levels of biomass and metabolite yields were associated with heterologous expression of VHb. For instance, improved biomass, spore, and protease production by the filamentous fungus *Paecilomyces lilacinus* and increased yields of total flavones and exopolysaccharides by *Phellinus igniarius* were obtained in SmF when engineering these fungi with VHb (Zhang et al. 2014; Zhu et al. 2011). Also, expression of the VHb in *Aspergillus niger* resulted in advantageous effects on the physiology of this fungus under oxygen-limiting conditions (Hofmann et al. 2009). Similarly, the increased biomass and extracellular-enzymatic content of *A. sojae* vgb + in our fermentation showed that overall VHb improved the strain’s adaptability to the fermentation conditions. The maximum productivities of the transformed and untransformed strain between the fourth and ninth day of fermentation were in good agreement with recent SSF with the parental fungus (Heerd et al. 2014b, 2012; Mata-Gomez et al. 2014) and demonstrated thus an unaltered time shifting on enzyme productivity by VHb. The highest mycelial density observed during this period of maximum productivities indicates also the importance of this morphological stage for the production of proteins by *A. sojae* (Fig. 3).

Contrary to the increase of biomass, protease, and exo-pectinase content in SSF, endo-PG production did not improve with *A. sojae* vgb + but was rather slightly lower in comparison to its parental *A. sojae* wt strain, at least during the stationary phase of fermentation between the fourth and eighth day (Fig. 4c). Similarly, it was shown that heterologous expression of VHb in *E. coli* appears to affect expression of several of its native genes in a different manner, either positively or negatively (Roos et al. 2004). In agreement with the previous study, the transformed VHb in *A. sojae* vgb + did not affect the production of different extracellular protein equally but rather favored certain enzymatic activities. The higher exo- to endo-pectinase activities suggest that under the SSF conditions, exo-pectinase activities are more essential for *A. sojae* vgb+, possibly to increase its biomass content. The more energy invested in exo-PG and biomass content may be connected to a trade-off concerning endo-PG production. Extending the investigation to other enzymatic activities besides hydrolases on *A. sojae* vgb + may uncover novel potential applications of this fungus.

Even though VHb has been extensively assessed, to date there is not a comprehensive understanding of how its expression affects biological production. In recent applications of VHb on fungi and yeast, where beneficial effects on growth and enzyme levels have been demonstrated, it has been pointed out that the mechanism of VHb can be rather complex (Shen et al. 2012; Wang et al. 2014; Wu and Fu 2012; Zhang et al. 2014). The common conception is that under oxygen-limiting conditions, VHb is induced in order to bind the remaining oxygen and deliver it to the terminal respiratory oxidase(s) to maintain aerobic respiration at a high level under these conditions (Stark et al. 2011; Webster 1987). VHb may also take part in various steps of the respiratory chain as terminal electron acceptor by improving ATP production or showing peroxidase activity; hence, the beneficial effects of VHb expression are presumably the result of one or more of its activities (Isarankura-Na-Ayudhya et al. 2010; Liao et al. 2014; Stark et al. 2015). Knowing that *A. sojae* has an aerobic metabolism, it may be implied that VHb favored the flux of oxygen in the transformant *A. sojae* vgb + during the SSF conditions, making this fungal strain able to consume more solid substrate and, in turn, increase biomass and several of its enzymatic contents. te Biesebeke et al. (2006) described that improvements on cell growth and enzymatic yields in an *A. oryzae* strain expressing hemoglobin domains similar to VHb may be due to an improved hyphae capacity to penetrate solid substrates. However, preliminary microscopic observations (data not shown) evidenced no remarkable difference on hyphal penetration depth between *A. sojae* vgb + and *A. sojae* wt on fermented substrate. This indicates that the major contribution of VHb on the transformed fungus may lie in its metabolism rather than in its phenotype. Even though the exact VHb mechanism on *A. sojae* still needs to be shown, the present study demonstrated clearly that VHb had a positive effect on *A. sojae* metabolism in solid-state cultures. Future SSF studies with the transformant *A. sojae* vgb + will show whether VHb will also have the same effect on the strain’s metabolism at a reactor scale.

In summary, this study genetically improved *A. sojae* ATCC 20235 for SSF application using the adapted ATMT method to integrate *vgb* under control of the constitutive P*gpdA* into the genome of this fungus. The transformed fungus *A. sojae* vgb+  showed improved biomass, protease, and exo-pectinase production, while its endo-PG content appeared slightly diminished in comparison to its parental strain in solid-state cultures. The fungal transformants generated within this study are suitable candidates to be evaluated in SFF scale-up studies in bioreactors. Based on our results, this genetic engineering strategy may also enable further optimization of *A. sojae* as a microbial biomanufacturing platform for pectinolytic enzymes, e.g., by iterative cycles of mutagenesis.
